# Supplementary figures and images for: Human milk bacteria assembled into functionally distinct synthetic communities in infant formula differently affect intestinal physiology and microbiota in neonatal mini-piglets
Source: mSystems. 2026 Mar 31;11(4):e00106-26. doi: 10.1128/msystems.00106-26 (PMC13098267; doi:10.1128/msystems.00106-26)

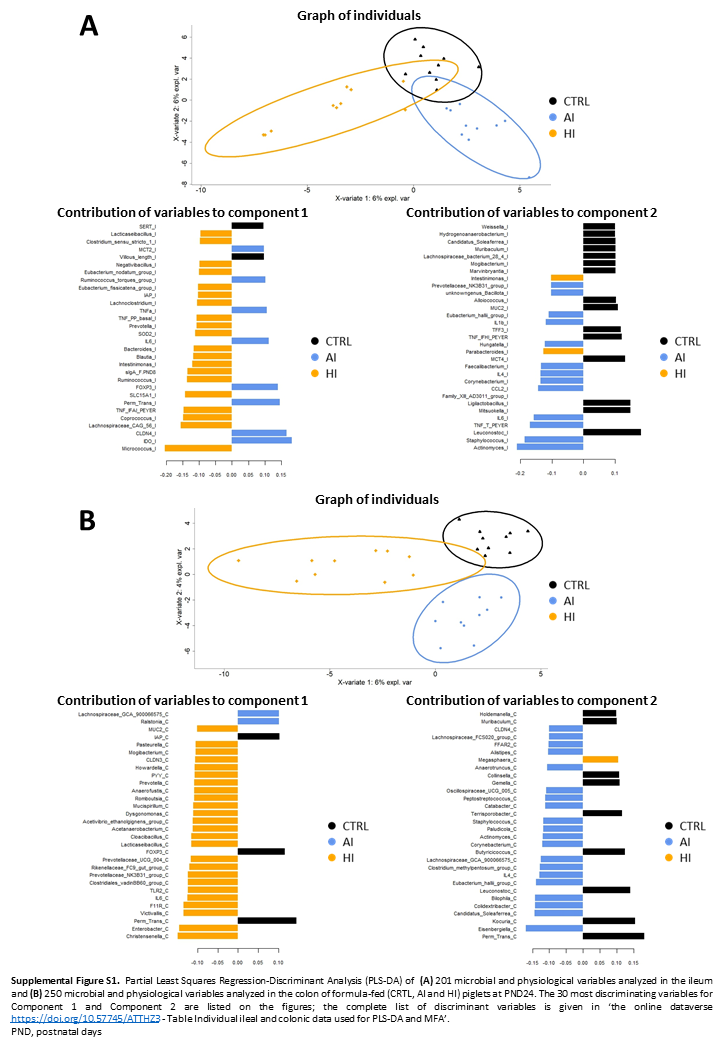

Supplement: Figure S1 — PLS-DA analysis of microbial and physiological variables in ileum and colon. [file msystems.00106-26-s0001.tif]

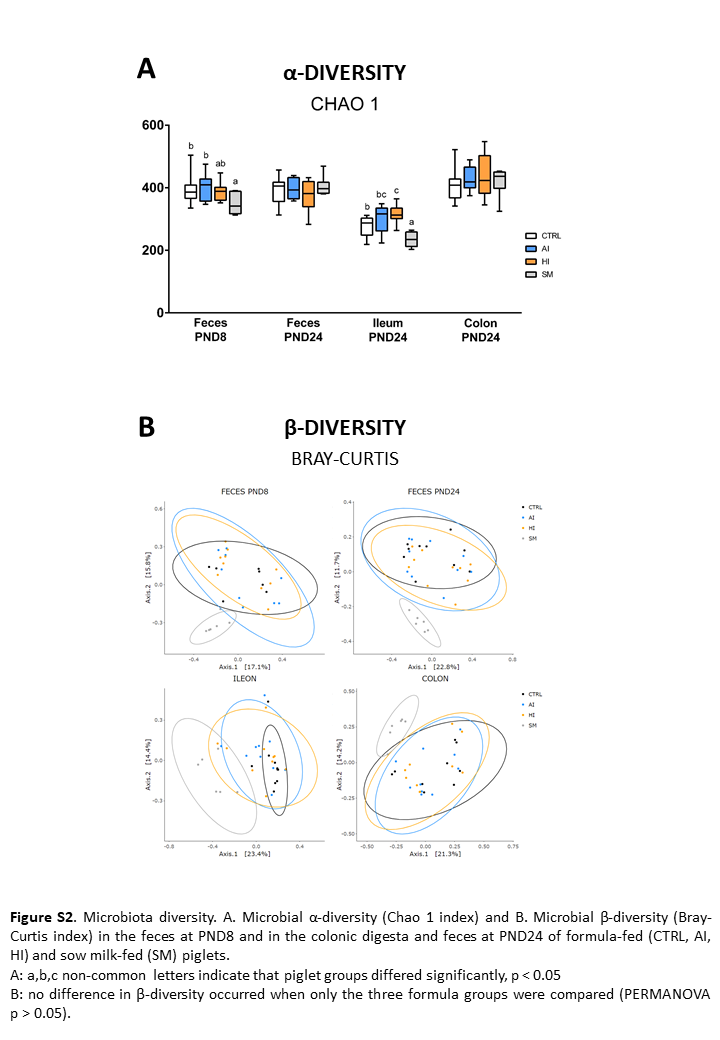

Supplement: Figure S2 — Microbiota diversity. [file msystems.00106-26-s0002.tif]

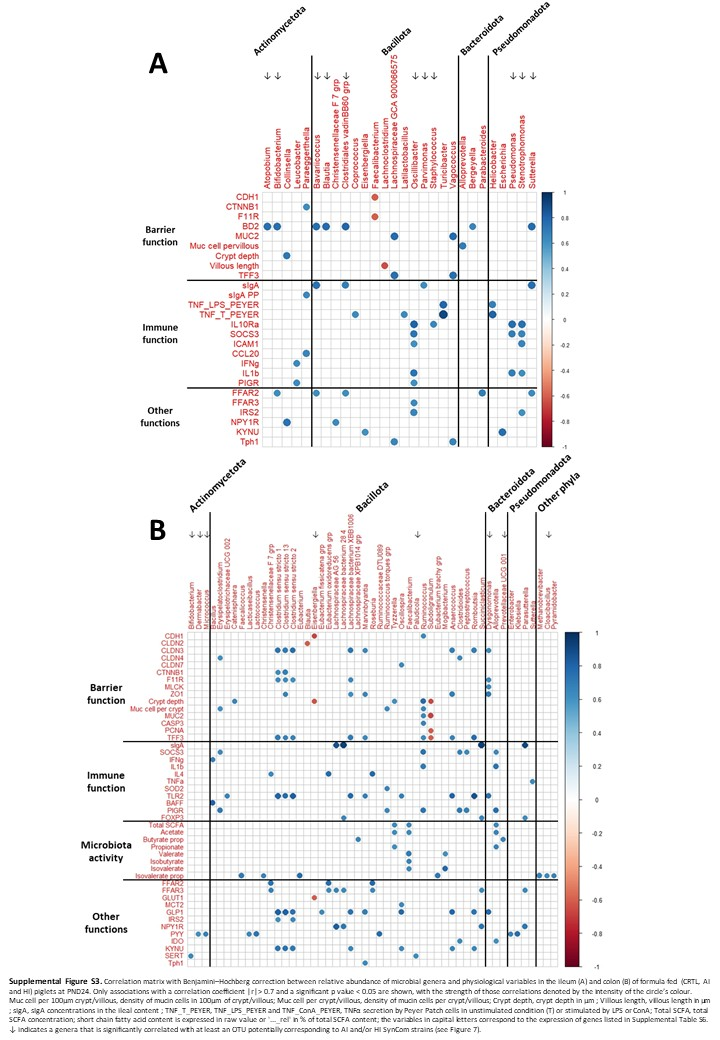

Supplement: Figure S3 — Correlation matrix between relative abundance of microbial genera and physiological variables in ileum and colon of formula-fed piglets at PND24. [file msystems.00106-26-s0003.tif]
