# Supplementary material for: Human milk bacteria assembled into functionally distinct synthetic communities in infant formula differently affect intestinal physiology and microbiota in neonatal mini-piglets
Source: mSystems. 2026 Mar 31;11(4):e00106-26. doi: 10.1128/msystems.00106-26 (PMC13098267; doi:10.1128/msystems.00106-26)
Supplement: Table S7 — Primers used in real-time PCR. [file msystems.00106-26-s0010.pdf]

Supplemental Table S7. Primers used in real-time PCR

Function	Function	Gene	Protein name	Sense (S) primer sequence	Anti-sense (AS) primer sequence
Barrier	Permeability	CDH1	Cadherin 1	catacacggccctcattatag	ctctggcagaagtccatattt
Barrier	Permeability	CLDN2	Claudin 2	gcactggcatcacccagtgt	gatgatacaggccaacgagg
Barrier	Permeability	CLDN3	Claudin 3	atcggcagcagcattatcac	acactttgcactgcatctgg
Barrier	Permeability	CLDN4	Claudin 4	aggagagacgcttcaatcgg	gtccagacacctgaacaccg
Barrier	Permeability	CLDN7	Claudin 7	tatgagtttggccctgccatc	ccttggcagaattgggcttag
Barrier	Permeability	CTNNB1	β-catenin	aggctcttgtgcgtactgtc	agagcacagatggcaggttc
Barrier	Permeability	F11R	Junctional adhesion molecule-A (JAM-A)	aatcagtgttccctcctctgctac	acggttgctcttgggctct
Barrier	Permeability	MLCK	Myosin light-chain kinase	tcggtgttgggcagtaagaaa	tgacattcaaaggctcagcatt
Barrier	Permeability	OCLN	Occludin	acgagctggaggaagactggatc	cccttaacttgcttcagtctattg
Barrier	Permeability	ZO1	Tight junction protein 1-Zonulin 1	aggcgatgttgtattgaagataaatg	tttttgcatccgtcaatgaca
Barrier	Defense	BD2	Beta defensin 2	ttgctgctgctgactgtctgcc	ttggccttgccactgtaacagg
Barrier	Defense	IAP	Intestinal Alkaline Phosphatase	ctaaaggggcagatgaatgg	cacctgtctgtccacgttgt
Barrier	Defense	MUC1	Mucin 1	gtgccgacgaaagaactg	tgccaggttcgagtaagag
Barrier	Defense	MUC2	Mucin 2	ggctgctcattgagaggagt	atgttcccgaactccaagg
Barrier	Apoptose	CASP3	Caspase 3	tgtgtgcttctaagccatgg	agttctgtgcctcggcag
Barrier	Proliferation	PCNA	Proliferating Cell Nuclear Antigen	gcagatgtaccccttgttgtagagt	atcttcgatcttgggagcca
Barrier	Proliferation	TFF3	Trefoil factor 3	agtatgtgggcctgtcggcgaa	atgctggagtcgaagcagcag
Endocrine	Endocrine	CHGA	Chromogranin A	aagtgcatcgtcgaggtcat	ttctgctgatgggacctctc
Endocrine	Endocrine	GCG	ProGlucagon Precursor	gctgatggtggcgatcttgt	tcccagctcttccgaaactc
Endocrine	Endocrine	GLP1R	Glucagon Like Peptide 1 receptor	tacttctggctgctggtggag	accccagcctatgctcaggta
Endocrine	Endocrine	IRS2	Insulin Receptor Substrate 2	ccgtgtcctctcccaaagtg	tgcgacccaatctcgatgtc
Endocrine	Endocrine	NPY1R	Neuropeptide Y Receptor Y1	tggcagagcaggaacaagag	tgtctctcattttgtccatcatgttgt
Endocrine	Endocrine	PYY	Peptide YY-Peptide Tyrosine Tyrosine	tcttctctccaaactgctcttcc	agatcggtgtattagcatatctccc
Immune system	Anti-inflammatory	BAFF	B-cell activating factor	gcatgccactgtctgcaatc	gagagcagctccattcaaag
Immune system	Anti-inflammatory	IL10	Interleukin 10	cggcgctgtcatcaatttctg	cccctctcttggagcttgcta
Immune system	Anti-inflammatory	IL10Ra	Interleukin 10 Receptor Subunit Alpha	tgcattctccacggcattct	cttcttggcagagtggaggg
Immune system	Anti-inflammatory	SOCS3	Suppressor Of Cytokine Signalling 3	tccagcatctctgtcggaaaac	ttaaagtggggcatcgtactgg
Immune system	Anti-inflammatory	TGFb1	Transforming Growth Factor Beta 1	aaaccctctatagcctctctgcgg	cgtggagctataccagaaatacagcaa
Immune system	Pro-inflammatory	CCL2	C-C Motif Chemokine Ligand 2	cctcatcctccagcatgaaggtctctgc	ggtggagtcaggcttcaaggcttcgg
Immune system	Pro-inflammatory	CCL20	C-C Motif Chemokine Ligand 20	gctcctggctgctttgatgtc	cattggcgagctgctgtgtg
Immune system	Pro-inflammatory	CX3CL1	C-X3-C Motif chemokine Ligand 1	gcagctcctagtccattac	caccattctgacccagaag
Immune system	Pro-inflammatory	IFNg	Interferon gamma	tggtagctctgggaaactgaatg	ggctttgcgctggatctg
Immune system	Pro-inflammatory	IL1b	Interleukin 1-Beta	gagctgaaggctctccacctc	atcgctgtcatctccttgcac
Immune system	Pro-inflammatory	IL4	Interleukin 4	cggtgacgacgtctttgct	cccggcagaaggtttgct
Immune system	Pro-inflammatory	IL6	Interleukin 6	ttctgtgactgcagcttatcc	gtcgaggctgtgcagattagt
Immune system	Pro-inflammatory	IL8	Interleukin 8	gctctctgtgaggctgcagttc	aaggtgtggaatgcgtatttatgc
Immune system	Pro-inflammatory	TNFa	Tumor necrosis factor alpha	ggttatcggcccccagaa	tgggcgacgggcttatc
Immune system	Pro-inflammatory	TNFaR1	Tumor necrosis factor alpha rcpt 1	gccacaaaggcacctaccta	gacatttcactccggcactt
Immune system	Antioxidant activity	SOD2	Superoxide dismutase 2	cctacgtgaacaacctgaac	gatacagcggtcaacttctc
Immune system	Treg pathway	FOXP3	Forkhead box P3	ctgacaagggttcctgctgt	gaaatctgggaacgtgctgt
Immune system	Receptor/cellular signaling	ICAM1	Intercellular Adhesion Molecule 1	cacaggccgccactaacaa	ggttccattgatccaggtcttg
Immune system	Receptor/cellular signaling	MYD88	Myeloid Differentiation Primary Response Gene	gatacccagtttgtgcaggagatga	atcagagacaaccactaccatccgac
Immune system	Receptor/cellular signaling	PIGR	Polymeric Immunoglobulin Receptor	gtagctaaaacaaccacgcaagaa	aggcgtagaagaagcgtgtca
Immune system	Receptor/cellular signaling	TLR2	Toll-like receptor 2	gactggccggagaactacct	tgagcaggagcagcaggaa
Immune system	Receptor/cellular signaling	TLR4	Toll-like receptor 4	gccatcgctgctaacatcatc	ctcatactcaaagatacaccatcgg
Nutrient transporter	SCFA receptor/transporter	FFAR2	Free fatty acid receptor 2	ggctttccccgtgcagtac	tgaacacgacggtgcagtga
Nutrient transporter	SCFA receptor/transporter	FFAR3	Free fatty acid receptor 3	cgagtggagaccttacgtgttg	cttggaaccccgaggatga
Nutrient transporter	SCFA receptor/transporter	MCT1	Monocarboxylate Transporter 1	agccaaccactgcagacaaa	gtatttgcgtcacttgcccc
Nutrient transporter	SCFA receptor/transporter	MCT4	Monocarboxylate Transporter 4	catcacgggcttctcctacg	agccgatgccgaactcac
Nutrient transporter	Carbohydrate transporter	GLUT1	Glucose Transporter Type 1	gcttccagtatgtggagcaact	aagcaatctcatcgaaggtcc
Tryptophan pathways	Kynurenine pathway	IDO	Indoleamine 2.3-Dioxygenase 1	gcccatgacttacaagaacatggat	ttgaatagagtggggatcaccttga
Tryptophan pathways	Kynurenine pathway	KYNU	Kynureninase	tcaagcctacaaccaaacggt	agctgcgactcaacagcata
Tryptophan pathways	Serotonin pathway	SERT	Serotonin transporter	gccagtaccaccgaaatgga	gtagaaggcgatgacgcaga
Tryptophan pathways	Serotonin pathway	TPH1	Tryptophan hydroxylase 1	gagcttcagaagaggctgttcaa	catagaccaaactccacagtgaaga
House keeping gene	House keeping gene	HPRT1	Hypoxanthine Phosphoribosyltransferase 1	tacctaatcattatgccgaggattt	agccgttcagtcctgtccat
House keeping gene	House keeping gene	PGK1	Phosphoglycerate Kinase 1	agataacgaacaaccagagg	tgtcaggcatagggatacc
House keeping gene	House keeping gene	RPL4	Ribosomal Protein L4	aggaggctgttctgcttctg	tccagggatgtttctgaagg
House keeping gene	House keeping gene	YWHAZ	Tyrosine 3-Monooxygenase/Tryptophan 5- monooxygenase Activation Protein Zeta	atgcaaccaacacatcctatc	gcattattagcgtgctgtctt

The primer were designed based on *sus scrofa* published nucleotide sequences
